# Deletion of Interleukin-1β Converting Enzyme Alters Mouse Cardiac Structure and Function

**DOI:** 10.3390/biology13030172

**Published:** 2024-03-07

**Authors:** Gohar Azhar, Koichiro Nagano, Pankaj Patyal, Xiaomin Zhang, Ambika Verma, Jeanne Y. Wei

**Affiliations:** Donald W. Reynolds Department of Geriatrics, Institute on Aging, University of Arkansas for Medical Sciences, Little Rock, AR 72205, USAppatyal@uams.edu (P.P.); zhangxiaomin@uams.edu (X.Z.); averma@uams.edu (A.V.); weijeanne@uams.edu (J.Y.W.)

**Keywords:** caspase-1, cardiac hypertrophy, oxidative stress, MAPK, apoptosis

## Abstract

**Simple Summary:**

Interleukin-1 converting enzyme/caspase-1 is a proinflammatory caspase that have been implicated in the pathogenesis of cardiovascular disorders, including heart failure and cardiac ischemia. In contrast, the role of the complete deletion of caspase-1 in the aging heart has not been fully studied. In this study, we aimed to understand how a lack of caspase-1 would impact the hypertrophic and apoptotic response in the mouse heart. We analyzed the hearts of ICE knockout mice to test the hypothesis that caspase-1 plays a significant role in cardiac morphology and function. Our study provides evidence that caspase-1 is an essential element in maintaining cardiac heath in aging and eliminating it will lead to hypertrophy, apoptosis, and increased vulnerability to oxidative stress.

**Abstract:**

Interleukin-1β converting enzyme (ICE, caspase-1) is a thiol protease that cleaves the pro-inflammatory cytokine precursors of IL-1β and IL-18 into active forms. Given the association between caspase-1 and cardiovascular pathology, we analyzed the hearts of ICE knockout (ICE KO) mice to test the hypothesis that caspase-1 plays a significant role in cardiac morphology and function. We characterized the histological and functional changes in the hearts of ICE KO mice compared to the Wild type. The cardiomyocytes from the neonatal ICE KO mice showed an impaired response to oxidative stress. Subsequently, the hearts from the ICE KO mice were hypertrophied, with a significant increase in the left ventricular and septal wall thickness and a greater LV mass/body weight ratio. The ICE KO mice hearts exhibited irregular myofibril arrangements and disruption of the cristae in the mitochondrial structure. Proapoptotic proteins that were significantly increased in the hearts of ICE KO versus the Wild type included pErk, pJNK, p53, Fas, Bax, and caspase 3. Further, the antiapoptotic proteins Bag-1 and Bcl-2 are activated in ICE KO hearts. Functionally, there was an increase in the left ventricular epicardial diameter and volume in ICE KO. In conclusion, our findings support the important role of caspase-1 in maintaining cardiac health; specifically, a significant decrease in caspase-1 is detrimental to the cardiovascular system.

## 1. Introduction

Despite advancements in research and treatment, cardiovascular disease remains the leading cause of death in the United States and around the world [1,2]. Heart failure is often preceded by cardiac hypertrophy, in which the heart attempts to enhance its function, leading to thickening of the muscle in response to elevated left ventricular stress [3]. There is considerable evidence of the presence of pro-inflammatory cytokines and chemokines in patients and experimental animals with progressive ventricular dilation and heart failure [4,5]. A major factor that mediates inflammatory injury in the heart and neutrophil activation is interleukin-1β (IL-1β) [6]. Elevated IL-1β has been observed in a variety of cardiac injury syndromes with oxidative damage and immune-mediated injury [7]. In cardiac myocytes, an excessive increase in IL-1β is involved in inflammation, apoptosis, and the disturbance of contractile function [8,9,10]. Other pro-inflammatory cytokines that are upregulated include TNF-α, IL-6, and IL-18, constituting an intrinsic stress response to myocardial injury [11,12].

Caspases are cysteine-class proteases that cleave after aspartate residues and regulate cellular processes such as apoptosis and differentiation, as well as inflammatory responses [13]. One such, the well-studied caspase-1, also known as ICE (interleukin-1β converting enzyme), converts biologically inactive preforms of interleukin-1β and IL-18 into their bioactive forms [14,15]. Consistent with the abnormal expression of IL-1β in cardiovascular-associated morbidity, IL-18 is also associated with cardiovascular disease and pathological changes in the blood vessels [16,17]. In addition to activating cytokines, caspase-1 is involved in the inflammatory programmed cell death of cardiomyocytes [18]. In mice, targeted deletion of caspase-1 is associated with reduced postoperative mortality and less left ventricular dilation after myocardial infarction [19]. Conversely, transgenic mice with cardiac-specific overexpression of caspase-1 suffer from ischemic damage and develop severe cardiac enlargement [20]. Although cardiac hypertrophy is observed in caspase-1 knockout mice subjected to renal ischemia/reperfusion [21], the role of caspase-1 deletion in the mouse heart has not been fully investigated. 

Further, caspase-1 has proapoptotic effects in cardiomyocytes [22,23], inducing apoptosis primarily through the activation of caspase-3 [24]. Further, oxidative stress and reactive oxygen species (ROS) contribute to the development of cardiac hypertrophy [25], where they activate signaling kinases and transcription factors, such as mitogen-activated protein kinase (MAPK), which mediates inflammasome formation and the processes of the proinflammatory cytokines interleukin IL-1β and IL-18 via caspase-1 activation [26]. Further, caspase-1 is a critical regulator of cardiomyocyte programmed cell death in the aging heart; the aging heart is characterized by an upregulation of oxidative stress, apoptosis, fibrosis, left ventricle hypertrophy, diastolic dysfunction, and a decline in overall function [27,28,29]. Caspase-1 expression is increased in the aging heart [30], and cardiac aging alters the expression of specific cytokines; for instance, IL-1β is significantly upregulated in the heart of aged mice compared to young mice [31]. However, the complete ablation of caspase-1 in the aging heart has not been fully elucidated. Herein, we investigated the hearts of ICE KO mice to test the hypothesis that caspase-1 maintains cardiac health both at the morphological and functional levels. 

## 2. Materials and Methods

### 2.1. Animals

The mouse strain with IL-1β converting enzyme (ICE) knockout (KO) was obtained from BASF (BASF Corporation, Florham Park, NJ) [32]. We also verified the deletion of ICE in the ICE-KO mice using Western blot analysis (Figure S1). In the present study, male and female adult and aged ICE-KO and Wild-type mice were used. Neonatal mice from 2 to 3 days old were also used for the cardiomyocyte isolation. Three or more mice per group were used in all experiments. The animals were housed in the Division of Laboratory Animal Medicine facility at the University of Arkansas for Medical Sciences (UAMS) on ventilated cage racks at 22–24 °C, with humidity at 30–50%, and using a 14 h light/10 h dark cycle. Ethical approval for the study was granted by the Institutional Animal Care and Use Committee at UAMS, in compliance with the Public Health Service Policy on the Humane Care and Use of Laboratory Animals and the National Research Council’s Guide for the Care and Use of Laboratory Animals. The experiments in this study were conducted in accordance with the ARRIVE guidelines [33].

### 2.2. Echocardiography

We used the Hewlett-Packard Sonos 5500 ultrasound imaging system (Philips, Andover, MA, USA) equipped with a 10 MHz pulsed array transducer for transthoracic echocardiography. Mice at 10 months of age were anesthetized using an intraperitoneal injection of ketamine (80 mg/kg) and xylazine (4 mg/kg). All the procedures and measurements were carried out as previously described [34]. 

### 2.3. Heart Weight/Body Weight 

The body weights of the Wild-type and ICE KO mice (*n* = 5) at different age points of 2, 4, 6, 8, 10, 14, 18, and 24 months old were measured before echocardiography. After, the mice were euthanized, and their heart tissues were dissected and then weighed. The heart weight/body weight ratio was calculated as heart weight (mg)/body weight (g).

### 2.4. Histological Analysis 

The mice were anesthetized deeply with carbon dioxide and euthanized via cervical dislocation. The experimental groups contained both male and female mice (10 months old, 5 animals per group). After euthanasia, the pericardial cavity was opened with a longitudinal section from the base to the apex of the heart. The mouse heart tissues were dissected in PBS, placed in relaxing buffer (25 mM KCl in PBS), and fixed in 10% neutral-buffered formalin overnight at 4 °C, followed by PBS washes and transfer to different gradients of ethanol for further processing. The atrial segment was dissected from the ventricular segment, and paraffin-embedded ventricle tissues were sectioned at a thickness of 3–4 mm. The sections were de-paraffinized in xylene and rehydrated with ethanol for histological analysis. Hematoxylin and eosin (H&E) and Masson’s trichrome staining (Poly Scientific; Bayshore, NY, USA) were performed according to the manufacturer’s protocol. Briefly, sections were deparaffinized and hydrated. The sections were stained in Weigert hematoxylin for 10 min and rinsed with water. Then, the sections were stained in Biebrich scarlet acid fuchsin for 5 min, rinsed with water, then transferred into aniline blue solution for 10 min, and rinsed again with water. Then, the sections were placed in 1% acetic acid solution and dehydrated with alcohol. Images were taken using a Nikon ES400 microscope. Measurements of the cardiac myocyte size were performed as previously described [34]. At least ten fields were examined at random from each section of the heart (left ventricular free wall, septum, and right ventricular free wall), and in those fields, 100 cardiomyocytes with nuclear profiles were measured. The cardiomyocyte area (µm^2^) was measured for comparison. 

### 2.5. Electron Microscopy

The mice were euthanized as described above, and the left ventricle apex was immediately dissected (10 months old, 3 animals per group). Transmission electron microscopy was performed as previously described [35]. Briefly, the heart tissues were fixed in 2.5% glutaraldehyde and 4% formaldehyde solution overnight at 4 °C. After dehydration, the tissues were embedded in Epon-812 epoxy resin, and ultrathin (70 nm) sections were prepared, followed by uranyl acetate and lead citrate staining. All the samples were prepared, examined, and photographed using a transmission electron microscope at the UAMS histology core facility.

### 2.6. RNA Isolation and Northern Blot Analysis

Total RNA was isolated from the left ventricular tissue samples using the ULTRA-SPEC RNA isolation reagent (BIOTECX Laboratories, Houston, TX, USA) and subjected to Northern blot analysis as previously described [34]. Loading control hybridization was performed using a 28S DNA probe labeled with [32 P] dCTP using random primer extension labeling. The scanning values for cardiac α-actin, skeletal α-actin, myosin heavy chain beta (β-MHC), ANP, and BNP mRNA were normalized to 28S mRNA. The expression signal intensity was quantified using the Storm system (Molecular Dynamics). Significant differences between the relative amounts of RNA of the ICE KO and Wild-type mice were determined using a two-tailed Student’s *t*-test (unpaired) using the GraphPad Prism 9.3.1 software.

### 2.7. Western Blot 

The left ventricular heart tissue was homogenized in 4% SDS and centrifuged at 12,000× *g* for 10 min at 4 °C, and the supernatant was transferred into a fresh tube. The lysate protein concentrations were determined using the Pierce BCA Protein Assay Kit (Thermo Scientific, Rockport, IL, USA). Protein (50 µg) electrophoresis and further steps of Western blotting were performed as described previously [34]. The primary antibodies used for WB analysis were purchased from Santa Cruz and used at a 1:1000 dilution. The primary antibodies were ERK1/2 (sc-514302), p-ERK (sc-7383), JNK (sc-571), p-JNK (sc-6254), p38 (sc-728), p-p38 (sc-7973), p53 (sc-98), Bax (sc-7480), Bag-1 (sc-939), Bcl-2 (sc-783), Fas (sc-8009), caspase-3 (sc-7272), caspase-1 (sc-392736), and actin (sc-1616). An HRP-conjugated anti-rabbit or mouse secondary antibody was used as the secondary antibody (1:5000; Invitrogen, Waltham, MA, USA). The immunoreactive bands were visualized using chemiluminescence (ECL, GE Healthcare Amersham, Waltham, MA, USA). Images were captured using a ChemiDoc MP Imaging System (Bio-Rad, Hercules, CA, USA). Densitometric analysis was carried out using the ImageJ software (Version 1.54g, National Institutes of Health, Bethesda, MD, USA). Statistical analysis between the relative amounts of different proteins was determined using two-way ANOVA with the Bonferroni multiple comparisons test using the GraphPad Prism 9.3.1 software. 

### 2.8. Neonatal Cardiomyocyte Isolation

Neonatal cardiomyocytes were isolated from 2- to 3-day-old neonatal mice. The mice pups were anesthetized via indirect placement on ice for 15–20 min and then euthanized via cervical dislocation. Afterward, their ventricles were removed and washed 3 times with D-Hanks balanced salt solution at 4 °C and then minced and incubated with 0.1% collagenase (Worthington Biochem. Corp., Freehold, NJ, USA) and 0.1% trypsin (Sigma-Aldrich; St. Louis, MO, USA) for enzymatic digestion. Detailed methods of isolation were described previously [36].

### 2.9. DNA Synthesis in the Cardiac Fibroblasts

Cardiac fibroblasts were isolated from hearts from 10-month-old mice. The isolation protocol was previously described [36]. Briefly, three to five adult heart ventricles were isolated and minced into small chunks and digested with the digestion mixture (trypsin and collagenase). Eventually, the collected cells were plated in tissue culture plates coated with poly-L-lysine. Cardiac fibroblasts were grown in 24-well plates to an approximate density of 2000/cm^2^. After reaching sub confluency, the cells were made quiescent through incubation in serum-starved conditions (0.2%) for 48 h. After H_2_O_2_ treatment, the cells were grown for 24 h and pulse-labeled with [3H] thymidine (1 μCi/mL) for 1 h. Further, the thymidine incorporation into the DNA was measured as trichloroacetic-acid-insoluble radioactivity. 

### 2.10. Beat Rate Assay

Neonatal cardiomyocytes from the ICE KO and Wild-type mice were cultured in laminin-coated culture dishes. Their beats were routinely counted every 5 min. The spontaneous beating frequency was measured via manual visual counting of the cardiomyocyte contractions. A Nikon light microscope with a 10× objective was used, and 15–20 cells were chosen at random at 3 or more locations.

### 2.11. Oxidative Stress in the Neonatal Cardiomyocytes

We assayed the cell death in response to oxidative stress as described previously [36]. The neonatal cardiomyocytes from the ICE KO and Wild-type mice were stained with trypan blue after exposure to hydrogen peroxide. The ratio of the number of unstained cells to total cells was evaluated as the percent of viable cells. The cell injury induced by oxidative stress was assessed by measuring the lactate dehydrogenase (LDH) release in the medium using a Pierce LDH cytotoxicity assay kit (Thermo Fisher Scientific, Waltham, MA, USA). 

### 2.12. Apoptotic Nuclear Changes in the Neonatal Cardiomyocytes 

DAPI (4′,6-diamino-2-phenylindole) (D1306, Invitrogen) staining was used to evaluate the apoptosis of the neonatal cardiomyocytes. The neonatal cardiomyocytes were exposed to 50 µm and 100 µm concentrations of H_2_O_2_ for 30 min. After incubation, the cells were washed three times with PBS and permeabilized with 0.2% Triton X-100. Further, for nucleus staining, the cells were washed again with PBS and incubated with 1.0 μg/mL of DAPI and then visualized under an immunofluorescence microscope. Morphological changes were determined by taking 10 random images from different sites in the cell culture dish. The percentage of apoptotic cells with an altered nuclear morphology and nuclear shrinkage was determined in triplicate. 

### 2.13. Statistical Analysis

The experiments were performed at least 3 times, and the data shown are means ± standard deviation (SD) or standard error of mean (SEM). Differences between the 2 groups were analyzed using a 2-tailed Student’s *t*-test. One-way analysis of variance (ANOVA) followed by Tukey’s pairwise multiple comparisons test, or 2-way ANOVA with the Bonferroni multiple comparisons test, was used to compare multiple groups. Results with *p* < 0.05 were considered statistically significant. The statistical analyses were performed using Prism 9.3.1 (GraphPad Software, San Diego, CA, USA).

## 3. Results

### 3.1. Assessment of the ICE KO Cardiac Structure and Function Using Echocardiography 

**Figure 1:**
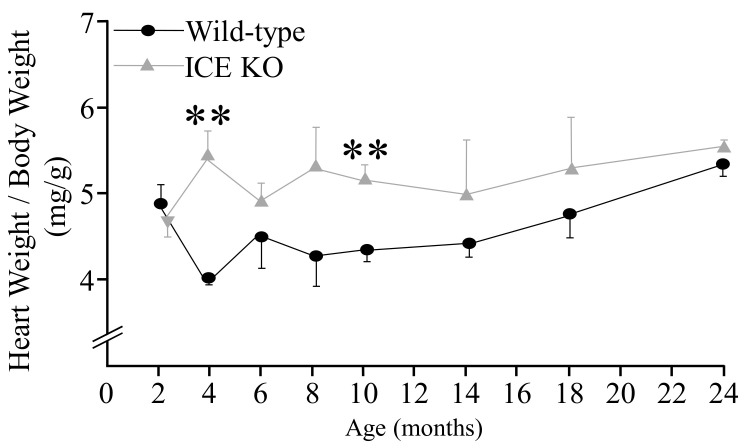
Heart weight normalized to body weight. Heart weight and body weight were measured at different time points (2, 4, 6, 8, 10, 14, 18, and 24 months old) for Wild-type and ICE KO mice. There was a significant increase in body weight and heart weight in ICE KO mice at 4 and 10 months of age. Data are presented as means ± SEM, *n* = 5, ** *p* < 0.001, 2-tailed Student’s *t*-test (unpaired).

Examination of the ICE KO mice at different age points revealed an increase in the heart-weight-to-body weight ratio (mg/g). ICE deletion increased the heart-weight-to-body-weight ratio (mg/g) in both the 4-month-old and 10-month-old mice compared to age-matched Wild-type mice (Figure 1 and Table 1). Echocardiography was then performed on 10-month-old mice (*n* = 5). Hypertrophy of the ICE KO hearts was evident due to a significant increase in the anterior wall (AW) and posterior wall (PW) thickness in both systole and diastole compared to the controls. The ICE KO hearts also showed a marked increase in epicardial volume in diastole. The left ventricular (LV) diastolic function was assessed by evaluating the ratios of E/A in Doppler tracings, where E is the peak velocity of early diastolic filling and A is the peak velocity of late filling associated with atrial contraction. The E/A ratios, and thus the diastolic function, were similar between the ICE KO and Wild-type mice. Although there was a tendency toward a slightly reduced cardiac index in ICE KO, the change was not significant, and left ventricular systolic function was preserved in the ICE KO mouse hearts (Table 1). These results suggest that the deficiency of caspase-1 alters the mouse heart structure and function.

### 3.2. ICE Deletion Induces Cardiac Hypertrophy, Fibrosis, and Mitochondrial Damage 

**Figure 2:**
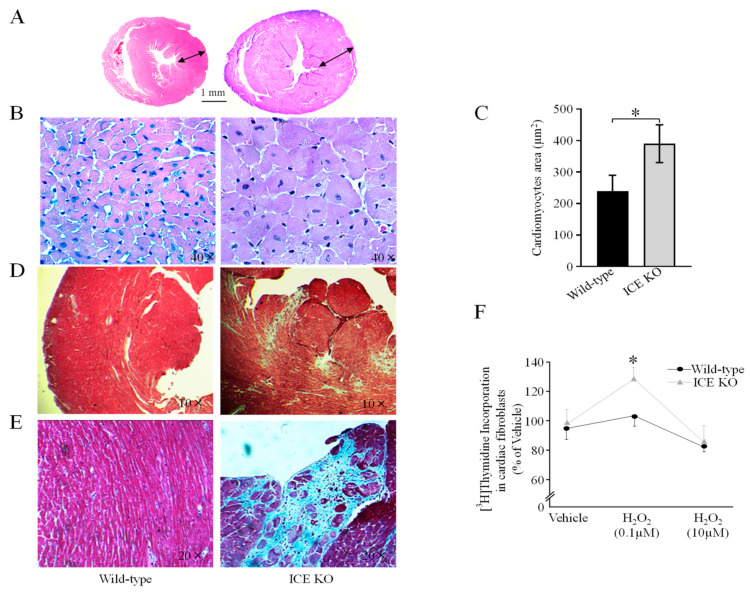
ICE deletion induces cardiac hypertrophy, fibrosis, and apoptosis. (**A**) Representative image of concentric LV hypertrophy at 10 months of age. Heart mass was increased in ICE KO mice and arrows indicate thickness of left ventricular wall. (**B**) High magnification (40×) view of cardiac muscle of 10-month-old mice with H&E staining. (**C**) The area of cardiomyocytes was analyzed statistically. The mean of cardiomyocyte area of ICE KO hearts was significantly greater than Wild-type. (**D**) Representative image of Masson trichrome-stained section at low resolution. (**E**) At higher magnification of Masson trichrome-stained section, there was greater deposition of collagen and fibrosis, as shown by the blue stain, in the 10-month-old ICE KO mice hearts. (**F**) The proliferation of the cardiac fibroblasts of adult ICE KO cardiac fibroblasts in response to hydrogen peroxide shows a significant increase at 0.1 μM dose. Significant difference between ICE KO and Wild-type mice (*n* = 5, * *p* ≤ 0.05) was determined using a two-tailed Student’s *t*-test (unpaired).

**Figure 3:**
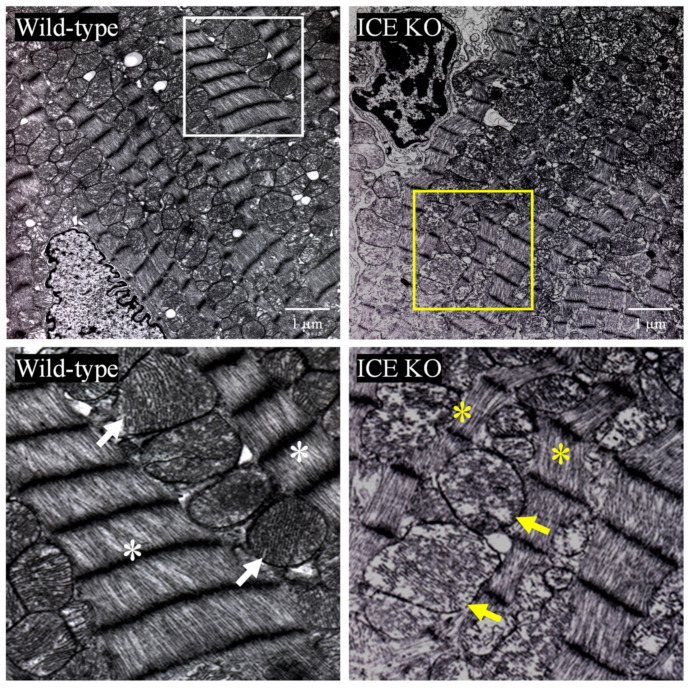
Detailed transmission electron microscopy of left ventricular tissue taken from Wild-type (**left panels**) and ICE KO mice (**right panels**). The white box (**upper left**) shows healthy myofibrils and mitochondria in Wild-type mice. The magnified section below with white asterisks shows regular arrangement of myofibrils, and the white arrows indicate very well-packed, regular cristae in the mitochondria. The yellow box (**upper right**) shows ICE KO with thinner myofibrils and degeneration of mitochondria. The magnified section of the yellow box below shows asterisks pointing towards shortened and disarrayed myofibrils and arrows indicating damaged mitochondria with loss of cristae. Animals from both groups were from 10-month-old mice. Scale bar is 1 µm. Images are representatives of 3 replicates.

To test the hypothesis that knocking out ICE contributes to the development of cardiac hypertrophy, we examined the hearts of 10-month-old mice using H&E staining. The hearts of the adult ICE KO mice were hypertrophied, with a significant increase in the left ventricular and septal wall thickness relative to the Wild-type hearts (Figure 2A,B). Histologically, the cardiomyocytes were larger in the hearts of the ICE KO mice compared to the controls (Figure 2C). Staining with Masson’s trichrome indicated greater deposition of collagen and fibrosis in the hearts from the 10-month-old ICE KO mice (Figure 2D,E, blue staining). We next examined whether apoptosis was involved in the pathogenesis of cardiac hypertrophy in the ICE KO mice. We isolated cardiac fibroblasts from the 10-month-old mice and tested their proliferation capacity in response to oxidative stress. The proliferation of the cardiac fibroblasts from the 10-month-old ICE KO mice increased significantly in response to oxidative stress at 0.1 μM H_2_O_2_ (Figure 2F). Further, we observed the ultrastructure of the ICE KO mouse heart sections using transmission electron microscopy (TEM). The ultrastructural analysis indicates shortened and disarrayed myofibrils, frequently fragmented cristae, and a loss of cristae in the hearts from the 10-month-old ICE KO mice (Figure 3). Taken together, our data suggest the caspase-1 deletion hypertrophied the mouse heart with significant fibrosis and further alternations in the mitochondrial structure. 

### 3.3. ICE-Deletion-Induced Expression of Markers of Cardiac Hypertrophy 

We further determined the gene expression of markers of cardiac hypertrophy, including alpha-skeletal actin (αSKA), alpha cardiac actin (ACTC1), beta-cardiac myosin heavy chain (β-MHC), and ANP and BNP genes. Both ANP and BNP expression indicated natriuretic peptide synthesis in the cardiac tissues. The mRNA expression in the left ventricles of the 10-month-old ICE KO and Wild-type mice was examined. Northern Blot analysis showed upregulation of the cardiac hypertrophic markers. In the left ventricle of the ICE KO mice, αSKA was increased 3.88-fold, ACTC1 was increased 1.47-fold, β- MHC was increased 7.41-fold, ANP was increased 3.72-fold, and BNP was increased 7.66-fold compared to that of the Wild-type mice (Table 2). These findings of the elevated gene expression of different markers of cardiac hypertrophy in the ICE KO mice validated our functional and histological observations.

### 3.4. ICE Regulates the MAPK Signaling Pathway and Apoptotic Proteins during Cardiac Hypertrophy

**Figure 4:**
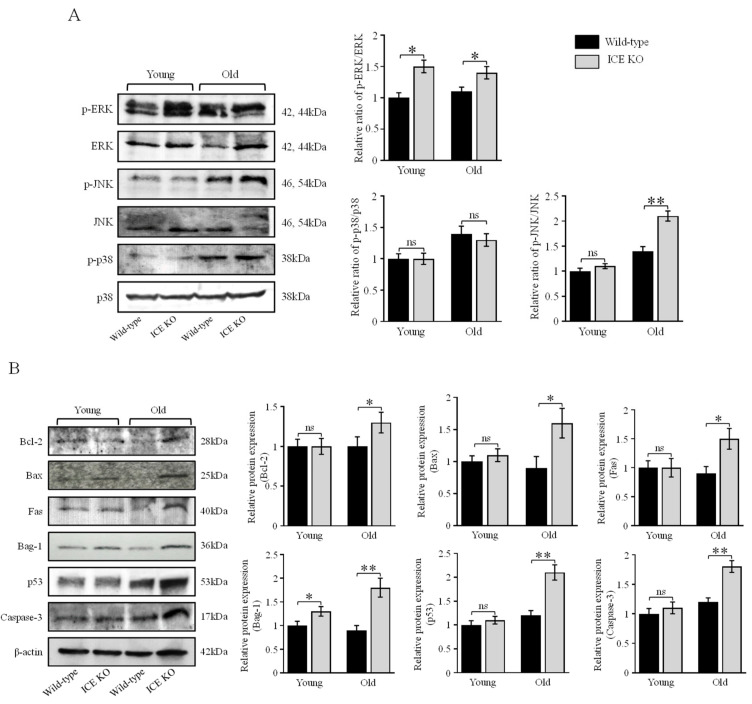
ICE deletion activates MAPK and apoptotic proteins. (**A**) Representative immunoblots of total phosphorylation of ERK, JNK, p38 in ICE KO and Wild-type mice hearts in young (4–6 months old) and aged mice (18–24 months old). Quantification of the relative changes in phosphorylation of ERK, JNK, and p38 into total protein was determined. (**B**) Caspase-3 and other proapoptotic and antiapoptotic protein expression was determined. Western blots show protein expression levels of Bcl-2, Bax, Fas, Bag-1, and p53. Molecular weight in kilo Dalton (kDa) are indicated. β-actin was used as a loading control. Relative protein expression was quantified. Data are expressed as mean ± SD (*n* = 3). Significant difference (* *p* < 0.05, ** *p* < 0.01, ns, *p* > 0.05,) between ICE KO vs. Wild type (analyzed using two-way ANOVA with Bonferroni multiple comparisons test).

MAPKs are involved in directing diverse cellular responses, including cellular proliferation, differentiation, mitosis, cell survival, and apoptosis (37). The activation of MAPK proteins plays an important role in the development of cardiac hypertrophy (38). Therefore, we further explored the effect of ICE deletion on MAPK signaling. We evaluated the protein expression in the left ventricular tissue from young (4–6 months old) and old (18–24 months old) ICE KO mice and age-matched Wild-type controls. We found that the MAPK protein pERK was significantly phosphorylated in the ICE KO mice versus the Wild type at young age, and the increase persisted in old age. Meanwhile, pJNK was significantly phosphorylated in the ICE KO mice in old age but not at a young age. The p38 MAPK protein did not exhibit any change in expression; however, it was increased in the mouse hearts in old age. Furthermore, the expression of the apoptotic inducer p53 was also elevated in old age in ICE KO mice hearts, but no change was observed at a young age. Caspase-3 is one the major mediators of apoptosis and has been implicated in producing cardiac hypertrophy. Our results showed that the caspase-3 expression was increased in old age in the ICE KO mice. The other pro and antiapoptotic proteins Bcl-2, Bax, and Fas were elevated in old age in ICE KO, whereas Bag-1 was found activated at both young and old age in the ICE KO mice hearts. (Figure 4A,B). Therefore, our data suggest the MAPK proteins phosphorylated with the deletion of the ICE gene in the mouse hearts might have contributed to activating proapoptotic signaling.

### 3.5. Functional Analysis of the ICE KO Neonatal Cardiomyocytes under Oxidative Stress

**Figure 5:**
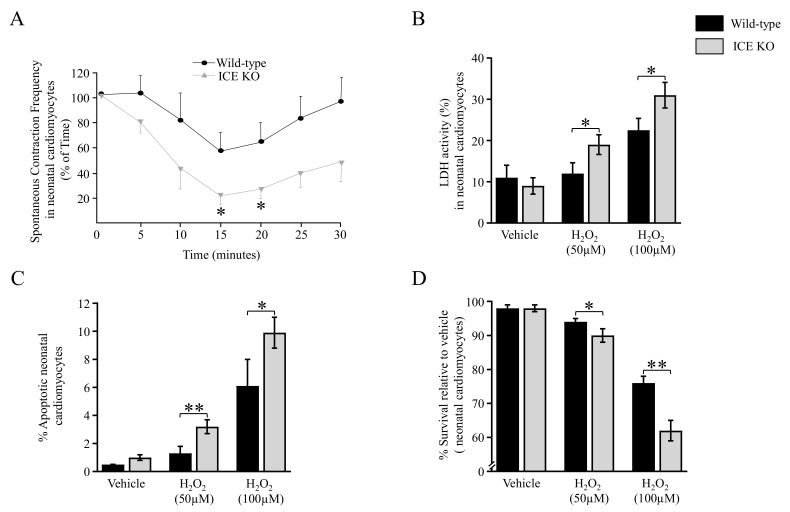
Oxidative stress increases in neonatal cardiomyocytes. (**A**) To evaluate the function of neonatal cardiomyocytes in response to oxidative stress, we assayed the spontaneous beat rate of cardiomyocytes. Line graph shows beat rate of neonatal cardiomyocytes in response to H_2_O_2_ (100 μM). There was a significant difference in contraction frequency at 15 and 20 min. (**B**) To evaluate cell injury induced by oxidative stress, we measured LDH release from cardiomyocytes exposed to hydrogen peroxide. Bar graph of LDH release from neonatal cardiomyocytes in response to H_2_O_2_ shows a significant increase in LDH activity at 50 μM and 100 μM concentrations of hydrogen peroxide. (**C**) Bar graph shows apoptotic nuclear changes in neonatal cardiomyocytes exposed to hydrogen peroxide. Morphologic changes in the nucleus were evaluated using nuclear staining with DAPI. There was a significant difference in the percentage of apoptotic cells between ICE KO and WT at different concentrations of H_2_O_2_ (50 μM and 100 μM). (**D**) The ratio of unstained cardiomyocytes using trypan blue to total number of cardiomyocytes was evaluated as the survival rate of neonatal cardiomyocytes when exposed to H_2_O_2_. There was a significant decrease in the survival of the ICE KO cardiomyocytes treated with H_2_O_2_ (50 μM and 100 μM). Data are expressed as mean ± SD (*n* = 3). Significant differences (* *p* ≤ 0.05; ** *p* ≤ 0.01) between ICE KO and Wild-type mice determined using a 2-tailed Student’s *t*-test (unpaired).

Oxidative stress is a key factor contributing to the development of cardiac hypertrophy. To further explore the role of ICE deletion in oxidative-stress-induced cardiomyocyte apoptosis, we examined the function of neonatal cardiomyocytes in response to oxidative stress. We measured the spontaneous beat rate of ICE KO neonatal cardiomyocytes in response to hydrogen peroxide (100 μM) and found a significant change in the contraction frequency at 15 and 20 min relative to the Wild type (Figure 5A). The cell injury induced by oxidative stress was quantified as the LDH released from the cardiomyocytes. There was a significant increase in the LDH released from the ICE KO neonatal cardiomyocytes in response to hydrogen peroxide (50 μM and 100 μM) (Figure 5B). In addition, hydrogen peroxide (50 μM and 100 μM) induced more apoptotic nuclear changes (Figure 5C), and cell death (Figure 5D), in the isolated ICE KO neonatal cardiomyocytes compared to the Wild type. Taken together, our findings suggest that the neonatal cardiomyocytes from the ICE KO mice were intolerant to oxidative damage and vulnerable to oxidative stress. Therefore, cardiomyocyte loss through apoptotic cell death may play an important role in the pathogenesis of cardiac hypertrophy in ICE KO mice. 

## 4. Discussion

Caspase-1 is known to activate proinflammatory cytokines [37], and decades of research have emphasized the role of caspase-1 in apoptosis, pyroptosis, necroptosis, and autophagy [38]. Caspase-1 not only regulates inflammatory cytokine maturation but also metabolism, unconventional protein secretion, and lysosomal function [39].

In general, this inflammatory caspase is an important regulator of the inflammatory response, which plays a critical role in defense against infectious diseases, as well as autoimmunity and in cancer [40]. Caspase-1 plays an important role in the regulation of cardiomyocyte biology. Several studies have investigated the role of caspase-1 in the pathogenesis of ischemic heart disease. Previous findings showed no abnormalities in histopathological evaluation of ICE KO mice hearts at 8 weeks of age [41]. In contrast, the role of the complete deletion of caspase-1 in cardiac function, particularly in adult and aging hearts, has not been fully investigated. Studies with cardiomyocyte-specific overexpression of caspase-1 demonstrated progression of heart failure [20], while endogenous deletion of caspase-1 was protective during myocardial-infarction-induced heart failure [19]. However, it was also demonstrated that caspase-1 induces programed cell death, thus allowing the elimination of abnormal cells and maintaining tissue homeostasis [42,43]. 

Our work highlights the importance of caspase-1 in maintaining normal cardiac function. The hearts from the ICE KO mice were hypertrophied and had increased levels of collagen, as well as increased apoptosis and sensitivity to oxidative stress. The chronic absence of caspase-1 appears to have a deleterious effect on the heart. Our findings demonstrate that the heart weight (normalized to body weight) was increased in ICE KO compared to the Wild type. Cardiac hypertrophy (measured by the left ventricular wall thickness) increased significantly in the hearts of the ICE KO mice, and this was corroborated using echocardiography. We then characterized the effect of cardiac hypertrophy on myocardial histology; in the ICE KO mice, there was an increase in the cardiomyocyte transverse cross-sectional area compared to the Wild type. There was also more fibrosis in the ICE KO hearts than in the Wild-type hearts. The increase in the gene expression levels of αSKA, ACTC1, and β-MHC in ICE KO further confirmed the cardiac hypertrophic response. The mRNA levels of ANP and BNP in the hearts of ICE KO were also upregulated, which shows hemodynamic overload and cardiac remodeling. Further functional data from echocardiography support the proposition that the absence of caspase-1 has deleterious effects similar to the overexpression of caspase-1.

A few studies have suggested that knocking out caspase-1 or using capase-1 inhibitors could provide cardioprotection by reducing inflammation-mediated damage [44,45,46]. However, our data suggest that the complete ablation of caspase-1 is deleterious, and optimal levels of caspase-1 may be essential to the normal functioning of the heart, as caspase-1 has a broad range of functions beyond inflammation [15]. In cardiomyocytes, the activation of the NLRP3 inflammasome triggers gasdermin-D and other apoptotic caspases to induce caspase-1-mediated cell death [47]; gasdermin-D is identified as a substrate of caspase-1 that mediates pyroptosis [48]. Conversely, in cardiac fibroblasts, caspase-1 regulates the maturation of IL-1β-mediated myofibroblast differentiation and further collagen synthesis [49]. Therefore, caspase-1-mediated inflammasome activation is cell-type-specific.

NLRP3 is a cytosolic multiprotein complex that mediates active IL-1β production and interacts with the precursor form of caspase-1, forming the NLRP3 inflammasome [50]. Further, MAPK signaling regulates the transcription of the NLRP3 inflammasome component [51]. Therefore, it is likely that the deletion of caspase-1 could have upregulated MAPK signaling. Further, several studies reported that p38 MAPK, ERK1/ERK2, and JNK play important roles in the development of cardiac hypertrophy [52]. ERK1/2 is activated in cardiomyocytes in response to stress [53], while ERK1/2 activation regulates cell survival and cell cycle progression in cardiac fibroblasts [54]. The JNK kinases have been implicated in cardiomyocyte growth and fibrosis, and JNK activation increases in the failing human heart and phosphorylates in cardiac hypertrophy [55]. Our data indicate the importance of caspase-1 in regulating MAPK signaling, as the phosphorylation levels of ERK1/2 and JNK in ICE KO were increased. Interestingly, the deletion of caspase-1 did not increase the phosphorylation of p38 in our study. It is possible that p38 might not have a key role in cardiac hypertrophy, as has been shown in other studies in which targeted activation of p38 in the heart did not produce cardiac hypertrophy [56]. The apoptotic inducer p53 was increased in the ICE KO mouse hearts. Furthermore, caspase-1 deletion also activated caspase-3 expression. Caspase-3 activation has been demonstrated to induce cardiomyocyte hypertrophy [24]. Further, the significant increase in the levels of the proapoptotic proteins Bax and Fas in the old-age ICE KO mice hearts suggests the presence of ongoing apoptosis. However, the increase in the antiapoptotic proteins Bag-1 and Bcl-2 suggest a possible compensatory antiapoptotic mechanism. These findings imply the deleterious effects of the caspase-1 deletion by ERK. JNK increased the phosphorylation of, as well as the subsequent activation of, caspase-3 and proapoptotic and antiapoptotic proteins. Therefore, caspase-1 possibly regulates MAPKs to modulate cardiac hypertrophy. Further, a recent study has shown transforming growth factor β-activated kinase 1 (TAK1) governs inflammation-induced cell death and hypertrophic growth [57]. TAK1 has been documented to regulate MAPKs such as JNK and p38 in the heart [58]. Thus, it is plausible that TAK1 along with MAPKs regulate the deleterious effects of caspase-1 deletion. 

The increased DNA fragmentation represents an acceleration of the biochemical process of apoptosis [59]. Our ICE KO results showed that a reduction in caspase-1 leads to increased apoptosis in the heart compared to the control Wild type. Our results showed that the ICE KO neonatal cardiomyocytes were more vulnerable to oxidative stress compared to the wild type, with a greater release of LDH and a significant reduction in the spontaneous beating rate. The electron microscopic appearance of the ICE KO mice hearts at 10 months of age demonstrated disruption of the cristae of the mitochondria and myofibril disorganization, which is generally visible with tissue injury. These findings suggest that knocking out caspase-1 has deleterious effects. 

## 5. Conclusions

In summary, our study suggests that the absence of caspase-1 has deleterious effects on cardiac structure and function in mice. Caspase-1 is an essential element for maintaining cardiac heath, and eliminating it can result in cardiac hypertrophy and increased vulnerability to oxidative stress. More research needs to be carried out to delineate the specific signaling pathways mediated by caspase-1 in the heart. Future in vivo studies will be needed in ICE KO mice to substantiate the deleterious effects of caspase-1 deletion.

## Figures and Tables

**Table 1:** Echocardiography. ICE KO mice (10 months old) were analyzed using echocardiography. ICE KO mice had significantly thicker heart walls and greater heart volumes than Wild-type mice. Data are expressed as mean ± SEM (*n* = 5). Significant difference (* *p* ≤ 0.05; ** *p* ≤ 0.01; *** *p* < 0.001) between ICE KO and Wild-type mice determined using a 2-tailed Student’s *t*-test (unpaired).

Parameters	Wild-Type	ICE-KO	*p* Value	
HR (bpm)	293.8 ± 74.3	216.7 ± 22.2	0.35	
SV (ml/beat)	33.15 ± 8.17	44.57 ± 10.91	0.42	
CI (ml/min/g)	348.9 ± 116.9	293.4 ± 95.6	0.72	
EnFS (%)	35.13 ± 16.20	44.57 ± 10.91	0.64	
MW FS (%)	20.30 ± 7.87	19.67 ± 1.81	0.94	
Peak E	0.58 ± 0.21	0.44 ± 0.05	0.53	
Peak A	0.30 ± 0.07	0.29 ± 0.04	0.81	
E/A	1.46 ± 0.09	1.45 ± 0.13	0.94	
PWs (mm)	1.02 ± 0.17	1.55 ± 0.14	0.04	*
PWd (mm)	0.58 ± 0.08	0.86 ± 0.09	0.008	**
AWs (mm)	0.98 ± 0.15	1.36 ± 0.05	0.0430	*
AWd (mm)	0.58 ± 0.07	0.94 ± 0.02	0.048	*
LVDs (mm)	2.37 ± 0.32	2.36 ± 0.21	0.97	
LVDd (mm)	3.65 ± 0.17	3.85 ± 0.31	0.58	
LV mass (g)	0.07 ± 0.01	0.13 ± 0.01	0.0003	***
BW (g)	28.0 ± 4.3	33.3 ± 0.5	0.25	
LV mass/BW	2.39 ± 0.29	3.91 ± 0.47	0.02	*
epi-D (mm)	4.80 ± 0.22	5.66 ± 0.21	0.02	*
epi-V (mm3)	111.3 ± 14.4	181.5 ± 20.8	0.02	*
Vols (mm^3^)	15.5 ± 10.4	13.4 ± 3.7	0.85	
Vold (mm^3^)	48.7 ± 6.8	58.0 ± 14.6	0.57	

**Table 2:** Ratio of mRNA levels in left ventricular heart tissue (ICE KO/Wild-type). Northern blot analysis was performed from total RNA isolated from ICE KO and Wild-type mice left ventricular heart tissue at 10 months old (*n* = 5, each). Data are expressed as mean ± SEM (*n* = 5). Significance difference (* *p* ≤ 0.05; ** *p* ≤ 0.01) between ICE KO and Wild-type mice determined using a two-tailed Student’s *t*-test (unpaired).

Genes	Fold Amount of mRNA Levels (ICE KO/Wild-Type)	*p* Value	
Cardiac α-Actin	1.47	0.006	**
Skeletal α-Actin	3.88	0.034	*
Myosin Heavy Chain Beta (MHC-β)	7.41	0.039	*
Atrial natriuretic peptide (ANP)	3.72	0.021	*
Brain natriuretic peptide (BNP)	7.66	0.036	*

## Data Availability

The raw data supporting the conclusions of this study will be made available by the corresponding author, without undue reservation.

## Supplementary Materials

The following supporting information can be downloaded at: https://www.mdpi.com/article/10.3390/biology13030172/s1, Figure S1: Western blot analysis of ICE deletion. Representative immunoblots of ICE from mouse heart left ventricular tissue. Western blots confirmed no ICE protein expression in ICE KO at young (4–6 months old) and old age (18–24 months old) mice heart tissues. β-actin was used as a loading control.
