# Supplementary material for: Deletion of Interleukin-1β Converting Enzyme Alters Mouse Cardiac Structure and Function
Source: Biology (Basel). 2024 Mar 7;13(3):172. doi: 10.3390/biology13030172 (PMC10968068; doi:10.3390/biology13030172)
Supplement: Supplementary file 1 [file biology-13-00172-s001.zip › biology-2779762-supplementary.pdf]

Deletion of interleukin-1β converting enzyme alters mouse cardiac structure and function

Gohar Azhar [1]*, Koichiro Nagano [1], Pankaj Patyal [1], Xiaomin Zhang [1], Ambika Verma [1], and Jeanne Y. Wei [1]

[1] Donald W. Reynolds Department of Geriatrics and Institute on Aging, University of Arkansas for Medical Sciences, Little Rock, AR 72205, USA.

Supplementary Figures

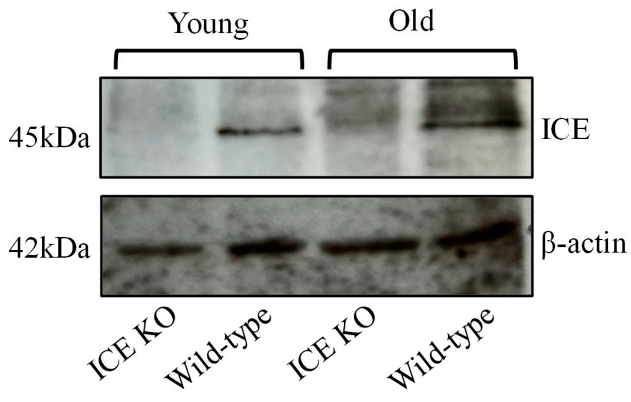

Figure S1. Western blot analysis of ICE deletion. Representative immunoblots of ICE from mouse heart left ventricular tissue. Western blots confirmed no ICE protein expression in ICE KO at young (4-6 months old) and old age (18-24 months old) mice heart tissues. β-actin was used as a loading control.
